# Caveolin-1 Promoted Collateral Vessel Formation in Patients With Moyamoya Disease

**DOI:** 10.3389/fneur.2022.796339

**Published:** 2022-04-26

**Authors:** Jinbing Zhao, Zhiqiang Yu, Yanping Zhang, Cheng Qiu, Guangxu Zhang, Lijiu Chen, Shengxue He, Jun Ma

**Affiliations:** Nanjing Comprehensive Stroke Center, Affiliated Nanjing Brain Hospital, Nanjing Medical University, Nanjing, China

**Keywords:** moyamoya disease, caveolin-1, collateral circulation, bypass surgery, stenosis

## Abstract

**Background:**

Caveolin-1 (Cav-1) plays pivotal roles in the endothelial function and angiogenesis postischemia. Moyamoya disease (MMD) is characterized by progressive artery stenosis with unknown etiology. We aim to determine whether serum Cav-1 levels of patients with MMD were associated with collateral vessel formation after bypass surgery.

**Methods:**

We studied serum Cav-1 levels of 130 patients with MMD (16 with *RNF213* p.R4810K mutation and 114 without *RNF213* p.R4810K mutation), 15 patients with acute stroke, and 33 healthy controls. Cerebral perfusion and collateral circulation were evaluated preoperation and at 6 months after operation using pseudocontinuous arterial spin labeling MRI (pCASL-MRI) and digital subtraction angiography (DSA), respectively. Endothelial expression of Cav-1 was verified in the superficial temporal artery (STA) wall of patients with MMD by immunofluorescence double staining. We also investigated whether overexpression of Cav-1 affects cell migration and tube formation using human microvascular endothelial cells (HMECs).

**Results:**

The serum Cav-1 level of patients with MMD intermediated between the stroke group and healthy controls and it was enhanced after the bypass surgery (681.87 ± 311.63 vs. 832.91 ± 464.41 pg/ml, *p* = 0.049). By 6 months after bypass surgery, patients with MMD with better collateral compensation manifested higher postoperative/preoperative Cav-1 ratio (rCav-1) than bad compensation patients. Consistently, cerebral blood flow (CBF) determined by pCASL-MRI (nCBF_MCA_ ratio) was positively in line with rCav-1 ratio (*r* = 0.8615, *p* < 0.0001). Cav-1 was expressed in the endothelial cells of the STA vessels of patients with MMD. Overexpression of Cav-1 by plasmid transfection in HMECs promoted tube formation and cell migration.

**Conclusion:**

This study indicated that Cav-1 may be a potential driver to promote angiogenesis and collateral formation after bypass surgery in patients with MMD, providing a better understanding of MMD pathophysiology and potential non-surgical targets of MMD.

## Introduction

Moyamoya disease (MMD) is a chronic progressive cerebrovascular disease with bilateral or unilateral distal internal carotid artery (ICA), middle cerebral artery (MCA), and anterior cerebral artery (ACA) stenosis or occlusion, accompanied by the formation of hazy vessels at the base of the brain. Owing to the stenosis of ICA, patients typically present with transient ischemia attack and infarction, while aberrant formation of basicranial hazy vessels leads to cerebral hemorrhage. In clinic, bypass surgery is a main option to restore cerebral perfusion for MMD (1), although it entails the possibility of perioperative complications and long-term insufficient collateral growth. Currently, there is no pharmaceutical treatment to prevent MMD progression (2). In theory, the outgrowth of preformed collateral vessels is the most important endogenous rescue system to cope with the chronic cerebral hypoperfusion (3). Therefore, the concept of therapeutic stimulating collateral vessel growth is supposed to be an effective remedy for patients with MMD.

Caveolin-1 (Cav-1) is a small concave protein, particularly abundant in endothelial cells, smooth muscle cells, fibroblasts, and epithelial cells (4). Cav-1 may promote angiogenesis and collateralization in ischemic stroke (5) and limb ischemia (6). On the other hand, Cav-1 recruited endothelial progenitor cells from the bone marrow to circulation, creating favorable conditions for vasculogenesis (7). Therefore, Cav-1 is predicted to be a possible target of MMD. Recent studies demonstrated that serum Cav-1 level was positively associated with the ICA diameter of MMD, indicating that Cav-1 may take part in ICA stenosis and occlusion (8). The Ring Finger Protein 213 (*RNF213*) gene was identified as the strongest susceptibility gene for MMD in the East Asian countries such as Japan (9). Bang et al. showed that the presence of the *RNF213* variant was associated with Cav-1 level, showing that the *RNF213* mutant patients with MMD have lower Cav-1 levels (10). These studies aroused our interest in studying the role of Cav-1 in angiogenesis of MMD in Chinese population. However, there is still lacking studies concerning the association between serum Cav-1 and the collateral formation of MMD. Additionally, whether compensative collateral network formation and cerebral perfusion after bypass surgery are associated with serum Cav-1 is unknown.

Conventional angiography such as digital subtraction angiography (DSA) is the mainstay to visualize the degree of artery lumen stenosis and the collateral vessels of MMD. Additionally, territory-specific perfusion measured by cerebral blood flow (CBF) imaging also plays a determine role in disease severity and whether to choose bypass surgery or not. Pseudocontinuous arterial spin labeling (pCASL) is a non-invasive MR perfusion imaging technique, which is a feasible way to access CBF changes in patients with MMD (11, 12). pCASL with multiple postlabeling delay (PLD) could provide more accurate CBF measurements because of the severe intracranial arterial stenosis and occlusion.

In this study, we investigated serum Cav-1 levels preoperation and 6 months postoperation and correlated Cav-1 levels with collateral compensation and CBF, with the purpose to find a better understanding of MMD pathophysiology and potential non-surgical targets of MMD. Furthermore, we proved the expression of Cav-1 in human artery and the function of that in *in-vitro* experiment. We hypothesized that enhanced postoperative expression of Cav-1 could be positively correlated with collateral formation and cerebral perfusion.

## Methods

### Patients

We prospectively recruited 130 patients with MMD (16 patients with *RNF213* mutation and 114 patients without *RNF213* mutation), 15 patients with acute stroke, and 33 healthy controls from the Nanjing Brain Hospital and Nanjing Drum Tower Hospital between January 2016 and May 2021. The diagnosis of MMD was based on the distal ICA or MCA/ACA stenosis and a hazy network of basal collaterals evaluated by DSA. Enrolled criterions of patients with MMD were as follows: (1) Adult patients with MMD; (2) All the patients underwent DSA examination and met the diagnostic criteria recommended by the Japan Research Committee on the Pathology and Treatment of Spontaneous Occlusion of the Circle of Willis in 2012; and (3) A written consent signed by the patients for the use of serum samples for biomarker examinations and other clinical data including DSA and MRI data. Patients with surgical contraindications were excluded. All the enrolled patients underwent unilateral superficial temporal artery-middle cerebral artery (STA-MCA) anastomosis and encephalo-duro-myo-synangiosis (EDMS). For patients with MMD with ischemic symptoms, bypass operations were performed at least 1 month after acute ischemia onset; for patients with MMD with cerebral hemorrhage, bypass operations were performed at least 2 months after bleeding absorption. Therefore, serum taken from patients with MMD was 1 or 2 months after acute cerebrovascular event. The STA samples were collected during the surgery. In the operation, the STA should be trimmed to an adequate length before anastomosed to M4 cortical artery and the trimmed STA arteries were collected as samples. In the control group, patients with temporal epilepsy underwent anterior temporal lobectomy or selective amygdalohippocampectomy and the STA trunk or branches under the incision were collected as samples for the control group. Patients with age-matched ischemic stroke (7 days within stroke onset) and healthy controls were recruited. This study was conducted in accordance with the Declaration of Helsinki and approved by the Ethics Committee of Nanjing Brain Hospital. Each patient signed a written consent.

### Blood Biomarkers for Caveolin-1

Serum samples were collected uniformly on the second day after patient admission. Among 130 patients with MMD, 55 patients provided the serum preoperatively and 6 months postoperatively. The other 75 patients only provided the serum preoperatively. Peripheral fresh blood samples were drawn out in coagulation tubes and serum was collected after centrifugation with 1,500 g for 10 min. Serum Cav-1 was quantified using the Cav-1 ELISA Kit (Catalog No. E0214h; EIAAB, Wuhan, China) according to the manufacturer's instructions. All the samples were run in duplicate. All the ELISA plates were read at the absorbance of 450 nm using the Tecan Spark 10M Microplate Reader (Tecan, Switzerland).

### Identification of the Ring Finger Protein 213 Mutation

The *RNF213* mutation analysis was performed by PCR amplification. The genomic DNA was extracted from peripheral blood leukocyte using the DNA Purification Kit (Promega, Madison, USA). Specific primers were designed according to the genomic sequence data of the *RNF213*, which was obtained from the National Center for Biotechnology Information website (http://www.ncbi.nlm.nih.gov/). The primers were designed as follows: *RNF213* p.R4810K F: TCTCGCAGCCAGTCTCAAAG and *RNF213* p.R4810K R: AGAGGGAGGTGCTTTTCAGC.

Polymerase chain reaction was performed using the ABI PRISM BigDye Terminator Cycle Sequencing Kit and the products were analyzed by the ABI 310 Genetic Analyzer (Applied Biosystems, California, USA).

### Human Microvascular Endothelial Cells Cultures and Plasmid Transfection

Human microvascular endothelial cells (HMECs) were grown in Dulbecco's Modified Eagle's Medium (DMEM) with 10% fetal bovine serum (FBS) maintained at 37°C in 5% CO_2_ atmosphere. HMECs were transfected with Cav-1 overexpression plasmid or vector CMV-MCS-IRES-EGFP-SV40-neomycin by lipofectamine 3000 (Thermo Fisher Scientific, USA) according to the manufacturer's protocols. Plasmid mixtures were added to the HMECs and incubated at 37°C in 5% CO_2_ for 6 h and then altered to fresh DMEM/5% FBS media. 24 h later, transfection efficiency was verified by Olympus IX73. Then, transfected HMECs were used to the following experiments.

### Capillary-Like Tubule Formation Assay and Wound Scratch Assay

BD Matrigel Basement Membrane Matrix (Corning, USA) was diluted 1:1 in the cultured media. Then, 96-well plates were precoated with 50 μl diluted mixture per well for 30 min at 37°C. HMECswere transfected with Cav-1 or vector plasmids for 24 h as described above. Then, cells were digested with trypsin and seeded at a density of 15,000 cells per well in the endothelial cell medium (ECM) (ScienCell Research Laboratories, USA) in Matrigel-coated plates and incubated at 37°C for a period of 20 h. The formation of capillary-like tubular structures was captured using an Olympus IX70 microscope and assessed with Image J version 1.8.0 software. Wound scratch assay was performed on confluent layers of transfected HMECs. 24 h after plasmid transfection, cells were seeded into 24-well cell culture plate confluently and were incubated for 24 h to allow them to adhere. Migration was initiated by scratching wound with the 10 μl pipette tip followed by two washes with DMEM media. Images of migration were taken immediately and 14 h after scratch.

### Magnetic Resonance Images and Cerebral Blood Flow Measurements

Cerebral blood flow of patients with MMD was investigated using pCASL sequence of 3.0-Tesla MR scanner (Discovery MR750, GE Healthcare, USA). Each raw ASL scan had a 3-dimensional fast gradient and spin-echo readout module with background suppression. Besides, we applied different postlabeling delays (PLDs) of pCASL scan for more accurate evaluation of cerebral perfusion. The standard and long PLDs were set as 1,525 and 2,525 ms, respectively. Quantitative measurement of regional CBF values was acquired by a recommended equation (13). Regions of interest (ROIs) were drawn over the MCA territory (CBF_MCA_) at the level of basal ganglia and ventricular roof (14–16). ROI within cerebellum (CBF_Cbll_) was set as control because of its hemodynamic stability in MMD (17). Thus, normalized CBF ratios adjusted to the cerebellum (nCBF_MCA_ = CBF_MCA_/CBF_Cbll_) were calculated to minimize the known inhomogeneous perfusion effect.

### Clinical Evaluation Scales

Collateral formations were graded by two independent experienced neurosurgeons. Preoperative angiographic stages of MMD by DSA were categorized into I to VI according to Suzuki and Takaku's Scale (18). Matsushima Clinical scale represents the clinical manifestations of each patient (19). Briefly, Type I: Transient ischemic attacks (TIAs) or episodes of reversible ischemic neurological deficit (RIND) occurring less than twice a month and with a normal CT appearance. Type II: Episodes of TIA or RIND occurring at least twice a month, with normal CT findings and neurological examination. Type III: Repeated TIAs or RINDs, with low density areas present on CT or with permanent focal neurological deficits. Type IV: Permanent focal neurological impairment secondary to cerebral infarction, followed by recurrent TIAs or RINDs or rarely repeated episodes of cerebral infarction. Type V: Neurological deficits due to cerebral infarction, followed by repeated episodes of cerebral infarction. Type VI: Intracerebral hemorrhage and other symptoms.

Then, DSA follow-ups were scheduled at 6 months after surgery to evaluate collateral revascularization. Revascularization assessments were conducted according to postoperative angiographic grading, as described previously (20). Briefly, Level 0: No obvious neoangiogenesis was found; level 1: collateral formation covering less than one-third of MCA territory; level 2: one-third to two-thirds of MCA territory; and level 3: collateral formation from external carotid artery system covering more than two-thirds of MCA territory. Based on this criterion, levels 0 and 1 were further defined as “bad compensation,” while levels 2 and 3 were defined as “good compensation.”

### Immunofluorescence

The STA vessels were fixed in 4% paraformaldehyde for 24 h and sliced by Leica slicer. Cryostat sections were washed twice in phosphate-buffered saline (PBS) and blocked with 2% bovine serum albumin (BSA) for 1 h. Sections were incubated sequentially with the primary antibodies for double immunofluorescent staining. Staining for Cav-1 and CD31 was performed first at 4°C overnight with rabbit monoclonal antibody to caveolin-1 (1:200; Abcam) and mouse monoclonal antibody to CD31 (1:500; Abcam), respectively. Sections were then washed in PBS three times for 10 min each time, followed by incubation with 594-conjugated donkey antirabbit immunoglobulin G (IgG) (1:500; Invitrogen, USA) and 488-conjugated donkey antimouse IgG (1:500; Invitrogen, USA) at 37°C for 1.5 h in the dark. Finally, 4',6-diamidino-2-phenylindole (DAPI) was used to stain the cell nuclei for 2 min at room temperature. After washed 5 times with PBS for 5 min each time, the slides were mounted and covered. Images were examined using Olympus IX70 microscope (Tokyo, Japan).

### Western Blotting

Twenty four hours after transfection, HMECs were lysed for detecting the expression of Cav-1. Briefly, 50 μg protein extracted from cell lyses was loaded to each lane, separated by 12% SDS-polyacrylamide gel electrophoresis (SDS-PAGE) and transferred to polyvinylidene difluoride (PVDF) membrane. The membranes were blocked with 5% non-fat milk and incubated overnight at 4°C with primary antibodies [rabbit antihuman caveolin-1, 1:1,000 dilution, Abcam, USA; rabbit antihuman glyceraldehyde 3-phosphate dehydrogenase (GAPDH), 1:2,000 dilution, Abcam, USA] and then incubated for 2 h with secondary antibodies. Membranes were developed by enhanced chemiluminescence (ECL) system.

### Statistical Analysis

All the statistical analyses were performed with SPSS version 26.0 (IBM Incorporation, Chicago, USA) and GraphPad Prism version 8.0 (GraphPad Software, San Diego, USA). Qualitative parameters of the different groups were analyzed by the Student's *t*-test and one-way or two-way ANOVA. Categorical variables and non-Gaussian distribution data were assessed by non-parametric tests such as the non-parametric Spearman's correlation analysis. The receiver operating characteristic (ROC) curve was run for testing the sensitivity and specificity for serum Cav-1 at different cutoff values. All the data were expressed as mean ± SD and *p* < 0.05 was set as statistically significant difference.

## Results

### Characterizations of Enrolled Patients

**Figure 1:**
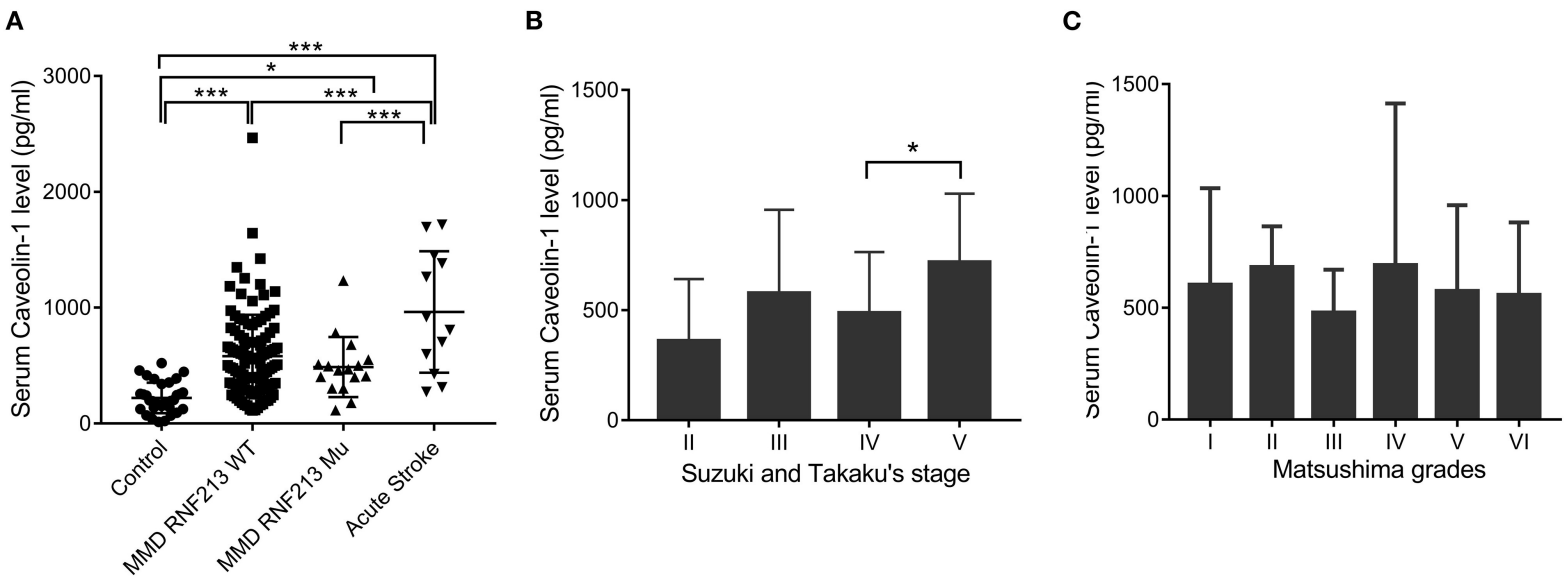
Serum caveolin-1 (Cav-1) levels in patients with moyamoya disease (MMD), acute stroke, and healthy controls. **(A)** Serum Cav-1 levels of patients with MMD [with or without the Ring Finger Protein 213 (*RNF213*) mutation], age-matched controls, and patients with acute stroke. **(B)** Serum Cav-1 levels of patients with MMD categorized by Suzuki and Takaku's stages. Stage V demonstrated higher Cav-1 levels than patients in stage IV. **(C)** Serum Cav-1 levels of patients with MMD categorized by the Matsushima grades. *p < 0.05, ***p < 0.001. WT, wild type; Mu, mutation.

A total of 130 patients with MMD, 15 patients with acute stroke, and 33 age-matched healthy controls were enrolled in this study. The baseline features of patients are given in Table 1. Generally, in the MMD group, 16/130 (12.31%) patients were the *RNF213* mutation. Patients with MMD frequently presented with ischemic stroke (46.92%), hemorrhage (27.69%), dizziness (32.30%), headache (25.38%), and transient ischemic attack (TIA) (9.23%). All the enrolled patients with MMD underwent bypass surgery after diagnosis. The postoperative complications frequently occurred during the first 2 weeks, including hemodynamic disorders (28/130), acute postoperative seizure (10/130), acute stroke (9/130), intracerebral hemorrhage (3/130), and wound complication (2/130). Most patients recovered during the follow-up period and only two patients suffered from moderate disability. The average Cav-1 levels of patients with MMD (without the *RNF213* mutation) was 581.56 ± 357.63 pg/ml, which was much higher than age-matched controls (221.52 ± 131.63 pg/ml, *p* < 0.001), but lower than the acute stroke group (891.27 ± 489.38 pg/ml, *p* < 0.001, Figure 1A). Additionally, there was no statistical difference of serum Cav-1 levels between the patients with the *RNF213* mutation and without the *RNF213* mutation (487.63 ± 259.70 vs. 581.56 ± 357.63 pg/ml, *p* = 0.7176, Figure 1A).

**Table 1:** The clinical properties of patients from healthy controls (CNT), MMD groups, and acute stroke group.

	**MMD** **(RNF213 WT)**	**MMD** **(RNF213 Mu)**	**Acute Stroke**	**Control**	***P***
Cases	114 (87.69%)	16 (12.31%)	15	33	
Age (y)	48.52 ± 10.57	41.06 ± 14.91	55.13 ± 16.47	46.67 ± 7.30	0.149
Sex (F/M)	52/62	8/8	6/9	16/17	0.861
Hypertension	48 (42.11%)	6 (37.5%)	12 (80%)	6 (18.18%)	<0.001
Diabetes mellitus	22 (19.30%)	4 (25%)	8 (53.33%)	0	<0.001
Hyperlipemia	4 (3.51%)	0	2 (13.33%)	0	0.079
**Initial presentation**
Headache	30 (26.32%)	3 (18.75%)	0	5 (15.15%)	0.731
Dizzy	38 (33.33%)	4 (25%)	0	5 (15.15%)	0.504
TIA	10 (8.77%)	2 (12.5%)	0	0	0.983
Stroke	51 (44.74%)	10 (62.5%)	15 (100%)	0	0.182
Hemorrhage	34 (29.82%)	2 (12.5%)	0	0	0.249
**Post-operative complications**
Acute stroke	8 (7.02%)	1 (6.25%)	-	-	1.000
ICH	2 (1.75%)	1 (6.25%)	-	-	0.328
Hemodynamic disorders	26 (22.81%)	2 (12.5%)	-	-	0.520
Seizures	10 (8.77%)	0	-	-	0.611
Wound complication	2 (1.75%)	0	-	-	1.000
Caveolin-1 (pg/ml)	581.56 ± 357.63	487.63 ± 259.70	891.27 ± 489.38	221.52 ± 131.63	

*MMD, moyamoya; RNF, Ring Finger Protein; WT, wild type; Mu, mutation; TIA, transient ischemic attack; ICH, intracerebral hemorrhage*.

### Serum Caveolin-1 Is Associated With Collateral Circulation Determined by Digital Subtraction Angiography

Among patients with MMD, Cav-1 level differs by angiographic features. Generally, there were 2, 74, 36, and 18 patients belonging to grades II, III, IV, and V by Suzuki and Takaku's Scale, respectively. Interestingly, patients in grade V, who were characterized with better spontaneous collateral formations, demonstrated higher Cav-1 levels than patients in grade IV (717.93 ± 311.79 vs. 487.74 ± 276.2 pg/ml, *p* < 0.05, Figure 1B). Cav-1 levels did not differ by clinical presentation scale—the Matsushima scale. The Matsushima scale VI reflects a group of patients with MMD with hemorrhage or other atypical symptoms, while the Matsushima scale I-V reflects patients with recurrent stroke or TIA (Figure 1C).

**Figure 2:**
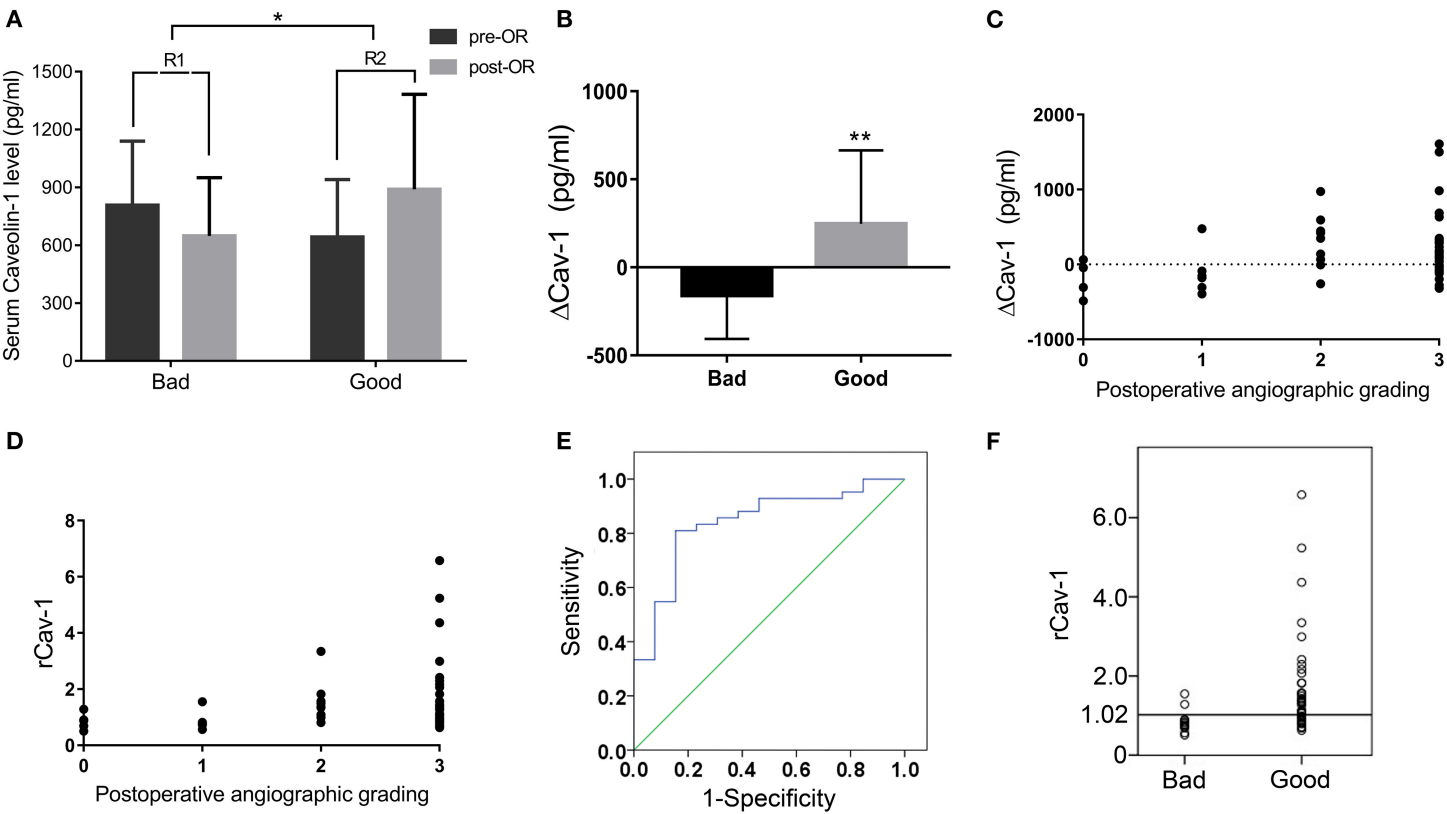
Serum Cav-1 increase after operation indicated good collateral circulation by digital subtraction angiography (DSA). **(A)** Preoperative and 6 months postoperative serum Cav-1 levels in both the bad and good compensation groups. The rCav-1 increased in the good compensation group (R2) and differed considerably from the bad compensation group (R1). **(B)** The ΔCav-1 value in the bad and good compensation groups, respectively. **(C,D)** ΔCav-1 and rCav-1 were positively correlated with the postoperative angiographic grading by the Spearman's correlation analysis (*r* = 0.3586, *p* = 0.0072; *r* = 0.3784, *p* = 0.0044, respectively). **(E,F)** The receiver operating characteristic (ROC) curve and cutoff value of rCav-1 in the bad and good compensation groups. The area under the curve was 0.841 (*p* < 0.0001), and the rCav-1 cutoff value was set at 1.02 with sensitivity at 71.4% and specificity at 84.6%. ΔCav-1: postoperative serum Cav-1 subtracted preoperative serum Cav-1; rCav-1: postoperative/preoperative Cav-1 ratio; *p < 0.05, **p < 0.01.

Preoperative and 6 months postoperative serum Cav-1 were detected in 55 patients with MMD and the baseline information is given in Table 2. By DSA grading, 5 patients were graded 0, 8 patients were graded 1, 9 patients were graded 2, and the rest 33 patients were graded 3. Therefore, there are 13 cases in the bad compensation group (DSA grades 0–1) and 42 cases in the good compensation group (DSA grades 2–3), respectively. We found that the Cav-1 levels increased obviously after surgery (preoperative 681.87 ± 311.63 vs. postoperative 832.91 ± 464.41 pg/ml, *F* = 3.981, *p* = 0.049), while the absolute Cav-1 levels did not show any statistical difference between the “bad compensation” and “good compensation” groups (*F* = 0.187, *P* = 0.666) by two-way ANOVA. Furthermore, the change of Cav-1 was analyzed. ΔCav-1 was defined as postoperative serum Cav-1 subtracted the preoperative serum Cav-1. We carefully assessed the revascularization by DSA after bypass surgery. Notably, good compensation patients presented much higher ΔCav-1 than the bad compensation group (*p* < 0.01, Figure 2B), so did postoperative/preoperative Cav-1 ratio (rCav-1, *p* < 0.05, Figure 2A). By the Spearman's correlation analysis, we observed that both the ΔCav-1 and rCav-1 were positively correlated with the collateral compensation (*r* = 0.3586, *p* = 0.0072; *r* = 0.3784, *p* = 0.0044, respectively, Figures 2C,D). Besides, the area under the ROC curve for rCav-1 was 0.841 (*p* < 0.0001). The rCav-1 cutoff value of 1.02 yields good sensitivity at 71.4% and specificity at 84.6% (Figures 2E,F). Therefore, higher postoperative/preoperative Cav-1 ratios indicated better collateral formation after bypass for patients with MMD.

**Table 2:** The clinical properties of 55 MMD patients with both preoperative and postoperative serum Cav-1.

	**Good compensation**	**Bad compensation**	***P***
Cases	42 (76.36%)	13 (23.64%)	
Age (y)	44.50 ± 12.21	52.92 ± 5.97	0.002
Sex (F/M)	22/20	6/7	0.695
Hypertension	13 (30.95%)	8 (61.54%)	0.098
Diabetes mellitus	7 (16.67%)	1 (7.69%)	0.725
Hyperlipemia	1 (2.38%)	0	1.000
**Initial presentation**			
Headache	11 (26.19%)	5 (38.46%)	0.616
Dizzy	14 (33.33%)	2 (15.38%)	0.370
TIA	4 (9.52%)	0	0.562
Stroke	16 (38.10%)	2 (15.38%)	0.235
Hemorrhage	14 (33.33%)	6 (46.15%)	0.610
**Post-operative complications**			
Acute stroke	1 (2.38%)	0	1.000
ICH	1 (2.38%)	0	1.000
Hemodynamic disorders	10 (23.81%)	3 (23.08%)	1.000
Seizures	3 (7.14%)	0	1.000
Wound complication	0	0	
*RNF213* mutation	6 (14.29%)	1 (7.69%)	0.883
DSA grading(0/1/2/3)	0/0/9/33	5/8/0/0	<0.001
**Caveolin-1 (pg/ml)**			
Preoperative	642.89 ± 298.40	807.81 ± 332.07	0.096
Postoperative	890.09 ± 493.13	648.16 ± 302.10	0.101
ΔCav-1	247.21 ± 64.41	−159.64 ± 68.65	0.002
rCav-1	1.67 ± 1.23	0.85 ± 0.28	0.021

*MMD, moyamoya disease; TIA, transient ischemic attack; ICH, intracerebral hemorrhage. RNF, Ring Finger Protein; ΔCav-1, post-operative serum Cav-1 subtracts the pre-operative serum Cav-1; rCav-1, post-operative/pre-operative Cav-1 ratio*.

### Serum Caveolin-1 Is Associated With Cerebral Blood Flow Determined by Pseudocontinuous Arterial Spin Labeling MRI

**Figure 3:**
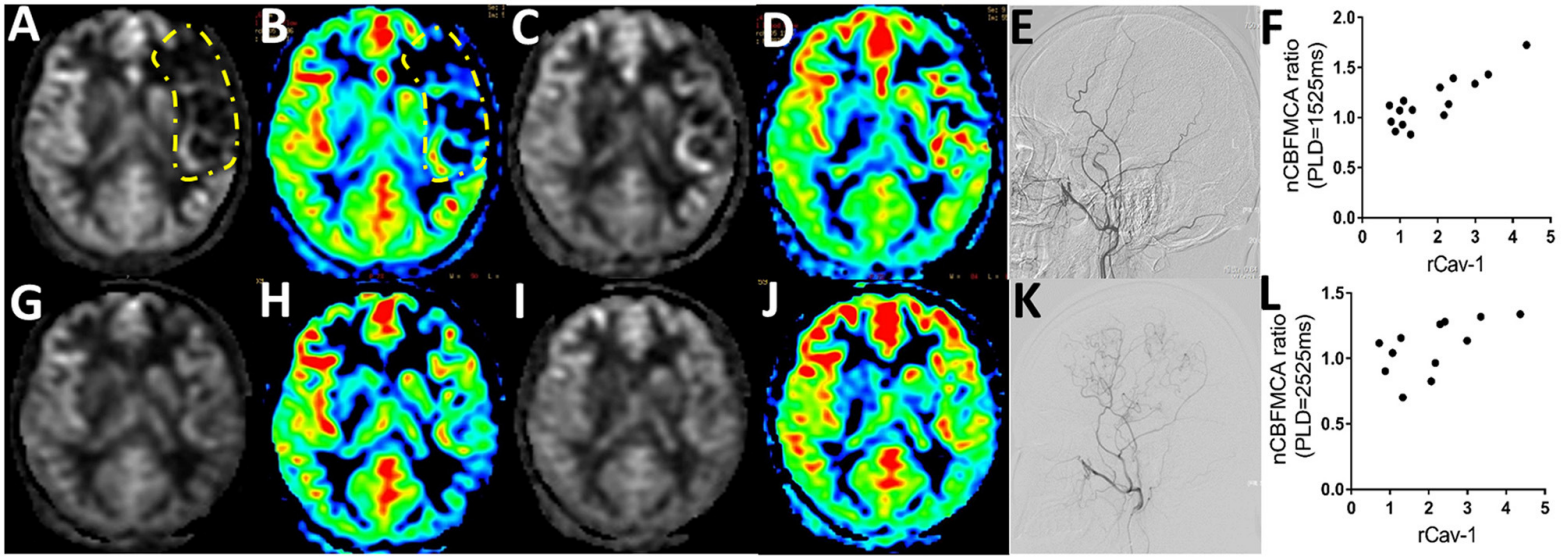
Serum Cav-1 was associated with cerebral blood flow (CBF) determined by arterial spin labeling MRI (ASL-MRI). **(A)** 24-year-old female patient was hospitalized for right hand numbness and diagnosed as MMD. The raw and color preoperative CBF maps by regular postlabeling delay (PLD) **(A,B)** and long PLD pseudocontinuous ASL (pCASL) imaging **(C,D)** demonstrated the hypoperfusion of middle cerebral artery (MCA) territory (circled by yellow lines). The CBF improved obviously in 6 months postoperative pCASL scanning with regular PLD **(G,H)** and long PLD **(I,J)**. The collaterals from the left external carotid artery before and after bypass surgery were shown in **(E,K)** by DSA. The rCav-1 had a strong positive relationship with the nCBF_MCA_ ratio in both the regular (*r* = 0.8615, *p* < 0.0001, **(F)**) and long PLD (*r* = 0.5915, *p* = 0.0428, **(L)**) by Spearman analysis. nCBF_MCA_ ratio, postoperative/preoperative nCBF_MCA_; nCBF_MCA_, CBF of MCA territory/CBF of ipsilateral cerebellum; rCav-1, postoperative/preoperative Cav-1.

The CBF is calculated as ml/100 g/min, indicating the volume of blood (ml) that circulates through 100 g brain parenchyma in 1 min. By pCASL-MRI, mean absolute CBF values in MCA territory of the anastomosed hemisphere were 33.70 ± 9.21 and 40.48 ± 9.16 ml/100 g/min at pre- and postoperative follow-up time points, respectively (*p* < 0.001). The pCASL-MRI changes of one typical patient with MMD underwent the left STA-MCA anastomosis and EDMS are shown in Figure 3. We observed the profound hypoperfusion signal of the left MCA territory from the raw and color CBF maps at PLD 1,525 ms (Figures 3A,B) and 2,525 ms (Figures 3C,D) before bypass surgery. By 6 months after bypass surgery, cerebral perfusion was improved by pCASL-MRI re-evaluation (Figures 3G–J), which was verified by DSA with abundant collateral formation in the left hemisphere (Figures 3E,K).

A total of 15 patients with MMD with detailed preoperative and postoperative clinical data (including serum Cav-1 levels, DSA, and pCASL images) were recruited for statistics. To minimize the inhomogeneous perfusion effect, we used normalized CBF ratio (nCBF_MCA_ ratio = Postoperative/Preoperative nCBF_MCA_) for further analysis. The rCav-1 had strong positive relationship with the nCBF_MCA_ ratio in both the regular PLD (1,525 ms, *r* = 0.8615, *p* < 0.0001) and long PLD (2,525 ms, *r* = 0.5915, *p* = 0.0428) by the Spearman's correlation analysis (Figures 3F,L). These trends supported higher serum Cav-1 ratios that represented improved CBF and cerebral perfusion after bypass surgery.

### Caveolin-1 Overexpression Promotes Endothelial Cell Migration and Capillary-Like Tube Formation

**Figure 4:**
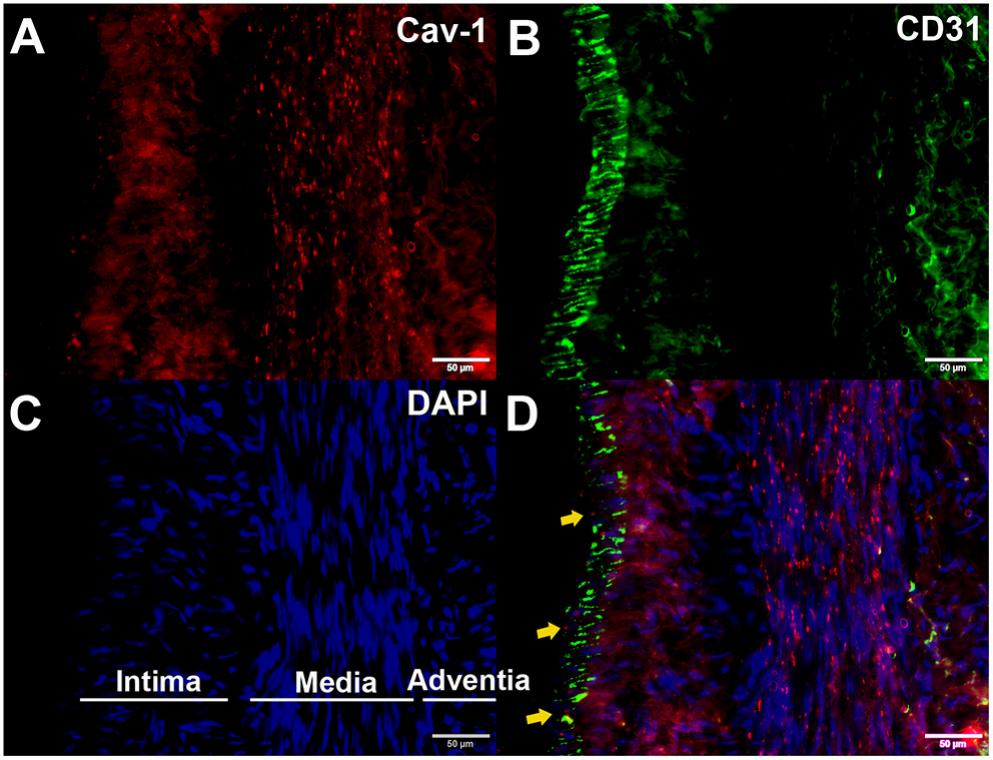
Enhanced expression of Cav-1 in the superficial temporal artery (STA) of patients with MMD. The STA samples from patients with MMD were stained with Cav-1 (red, **A**), CD31 (green, **B**), and 4',6-diamidino-2-phenylindole (DAPI) (blue, **C**). Cav-1 was mainly located in the intima and media of the STA (yellow arrows, **(D)**). Scale bar = 50 μm.

**Figure 5:**
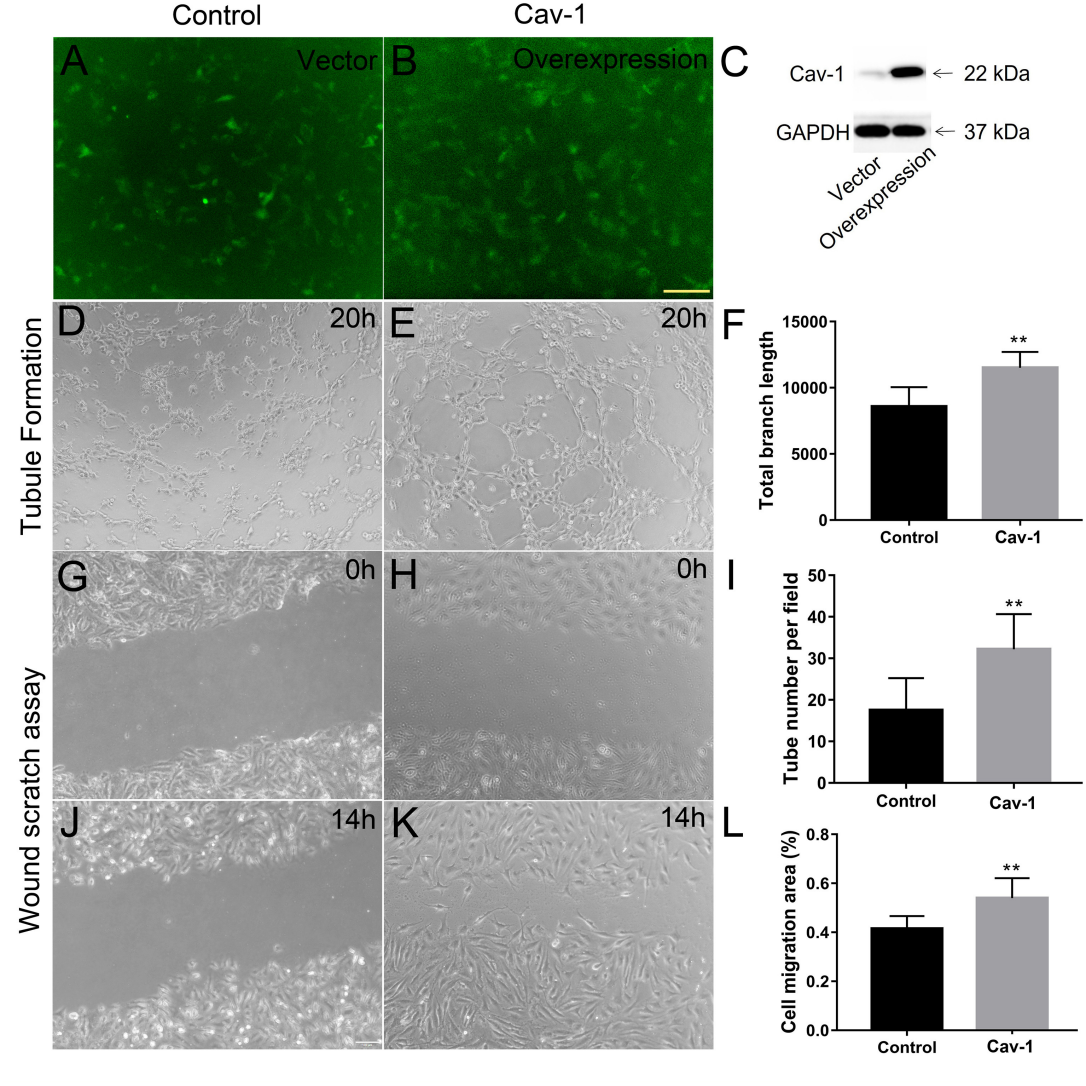
Cav-1 overexpression promotes endothelial cell proliferation and capillary-like tube formation. The cultured human microvascular endothelial cells (HMECs) were dealt with empty vector **(A)** and Cav-1 overexpression plasmid vector **(B)** for 24 h. before the following two assays (green signals represented successful transfection) and the enhanced expression of Cav-1 after transfection was confirmed by Western blotting **(C)**. The capillary-like tube formation assay **(D,E)** was evaluated at 20 h after HMECs reseeded and the total branch length and tube numbers in per field were calculated **(F,I)**. Wound scratching assay of HEMCs was assessed at 0 **(G,H)** and 14 h **(J,K)** after scratching by the 10 μl pipette tip for both the groups. The residual area after scratching was calculated to evaluate the cell migration ability **(L)**. **p < 0.01; scale bar = 100 μm.

We further determined whether endothelial cells could express Cav-1 in patients with MMD. The STA tissue samples of patients with MMD were stained with Cav-1 (Figure 4A), CD31 (Figure 4B) and 4',6-diamidino-2-phenylindole (DAPI, Figure 4C). As shown in Figure 4D, Cav-1 was mainly located in the intima and media of the STA. CD31 positive cells co-expressed Cav-1, indicating endothelial cells could express Cav-1 (Figure 4D). Then, we investigated Cav-1 functions in cultured HMECs. The cultured HMECs were dealt with empty vector (Figure 5A) and Cav-1 overexpression plasmid vector (Figure 5B) for 24 h before the following two assays. The green signals represented successful transfection and the enhanced expression of Cav-1 after transfection was confirmed by Western blotting (Figure 5C). Transfecting Cav-1 accelerated tubule formation, leading to a dramatic 1.83-fold increase in the number of capillary-like tubular structures (*p* < 0.01, Figures 5D,E,I). Consistently, tube branch length of the Cav-1 transfection group was 1.34-fold of that in the control group (*p* < 0.01, Figures 5D–F). Next, we assessed the effect of Cav-1 on endothelial cell migration by wound scratch assay. HMECs migration in the Cav-1 overexpression group and the control group was analyzed at 0 and 14 h after cell scratching. As shown in Figures 5G–L, HMECs in the Cav-1 overexpression group demonstrated reduced scratching gap compared to the cells in the control group (*p* < 0.01). The results demonstrated that Cav-1 promoted the migration of endothelial cells.

## Discussion

To summarize our findings, the serum Cav-1 level of patients with MMD intermediated between the stroke group and healthy controls and it was enhanced after the bypass surgery. Patients with MMD with better collateral compensation manifested higher postoperative/preoperative Cav-1 ratio (rCav-1) than bad compensation patients. The rCav-1 cutoff value of 1.02 yield good sensitivity (71.4%) and specificity (84.6%) in determining good or bad compensation. Consistently, CBF determined by pCASL-MRI (nCBF_MCA_ ratio) was positively in line with postoperative/preoperative Cav-1 ratio. Cav-1 could be expressed in the endothelial cells of the STA vessels of patients with MMD. Overexpression of Cav-1 by plasmid transfection in HMECs promoted tube formation and cell migration. This study indicated that Cav-1 could be a potential driver to promote angiogenesis and collateral formation of patients with MMD.

Moyamoya disease is an idiopathic cerebrovascular disease with unknown etiologies. Multiple factors involving genetic, inflammatory, and angiogenic aspects covalently contributed to MMD pathogenesis (2, 21). The *RNF213* has been confirmed to be a high susceptibility gene for MMD in East Asian populations. It is reported that p.R4810K mutation of the *RNF213* gene is about 13% in Chinese Han population (22) and 80% in sporadic patients with MMD of Japan (9, 23). Consistent with previous literatures, the incidence of the *RNF213* p.R4810K mutation is about 12.3% (16/130) in this study. One study from the Korea cohort demonstrated that the *RNF213* mutant patients with MMD were associated with lower serum Cav-1 levels (10), while another study of Chung (8) did not find any difference between the *RNF213* mutant and non-mutant groups, which is similar with the results of this study. This discrepancy may be caused by racial difference or statistical bias due to insufficient sample sizes. The *RNF213* global knockout mice demonstrated similar Cav-1 level with wild-type mice in endothelial cells of lung tissue (24). The correlation between the *RNF213* and Cav-1 does not come into consensus and needs further study. In our Chinese cohort, the serum Cav-1 level of patients with MMD intermediated between the stroke group and healthy controls. Our result is consistent with previous studies showing that serum Cav-1 level of patients with acute stroke is much higher than patients with MMD (8) and age-matched healthy controls (25). Whether the serum Cav-1 comes from is currently unknown. Studies from mouse ischemic stroke model demonstrated that Cav-1 immunoreactivity is significantly and specifically increased in the endothelial cells in the infarcted area 24 h post-MCA occlusion (MCAO). Serum Cav-1 is positively correlated with Cav-1 immunoreactivity of the brain (26). Accumulating evidence pointed out that Cav-1 was expressed in human brain microvascular endothelial cells and smooth muscle cells (8, 27). In this study, we observed the expression of Cav-1 in endothelial cells and smooth muscle cells in patients with MMD. Therefore, it is possible that serum Cav-1 may be derived from vessel wall. However, this hypothesis needs further investigation.

Although we observed the serum alternation of Cav-1 in patients with MMD, clinical significance of Cav-1 is still unknown. Preliminary study reported that patients with MMD with higher baseline Cav-1 level was positively associated with larger ICA diameter (8). MCA or ICA diameter was not a staging parameter in Suzuki and Takaku's grades. Suzuki and Takaku's grades are sensitive to categorize the collateral establishment. During the progression of Suzuki and Takaku's stages IV–V, abnormal smoking-like vessels begin to decrease, while good collateral circulation gradually develops. In this cross-sectional study, baseline serum Cav-1 levels in the stage V group were higher than that in the stage IV group, which provided a hint that Cav-1 may take part in the progression of collateral branch formation. Nevertheless, the level of Cav-1 among each grade may be a comprehensive result of multiple factors, including negative remodeling and collateral formation. Combined STA-MCA anastomosis and encephalo-duro-myo-synangiosis surgery (EDMS) is considered to be the optimal therapy for patients with MMD (1). All our enrolled patients received bypass surgery. This longitudinal study initially applied Cav-1 detection during the anastomosis follow-up period. It beared noting that patients with better Cav-1 expression indicated good collateral circulation compensation. Currently, DSA is still the “gold standard” for evaluating the severity of moyamoya angiopathy and the revascularization after bypass surgery (28). Among the promising non-invasive imaging options, ASL enables CBF evaluation by magnetically labeling the protons of inflows in feeding arteries. Pseudocontinuous ASL with multiple PLDs offers more accurate information compared to ASL with a single standard PLD (29, 30). With the application of DSA and pCASL with two PLDs, this study demonstrated that good post-/preoperative Cav-1 ratio was positively correlated with the establishment of collateral circulation and cerebrovascular perfusion improvement. The rCav-1 cutoff value of 1.02 yields good sensitivity (71.4%) and specificity (84.6%) in determining good or bad compensation after bypass surgery. PET/single-photon emission CT (SPECT) was considered to be a gold standard for estimating brain perfusion. However, those perfusion studies cannot be performed frequently due to the cost, contrast use, and radiation dose. ASL-MRI is a non-invasive and could be repetitively performed examination, which enables us to estimate brain perfusion frequently over a long-term follow-up period in MMD. Studies comparing SPECT and ASL-MRI addressed a strong correlation coefficient in patients with MMD (31). Recent studies in patients with steno-occlusive disease demonstrated that higher test-retest reliability led to better ASL sensitivity than SPECT for postoperative ΔCBF, suggesting that ASL-MRI may be better than SPECT for postoperative re-evaluation. Additionally, ASL with postlabeling delay of 2,333 ms presented higher reliability and sensitivity to ΔCBF (32). In this study, we evaluated CBF_MCA_ ratio (postoperative/preoperative nCBF_MCA_) in both the regular PLD (1,525 ms) and long PLD (2,525 ms), which was reliable and feasible. These findings indicated that serum Cav-1 in MMD could be a promising biomarker used to determine the collateral circulation after bypass surgery.

Besides MMD, serum level of Cav-1 has been reported in other cerebrovascular disease. In acute ischemic stroke, lower serum Cav-1 level is associated with the presence of microbleeds (33) and predicts symptomatic bleeding after thrombolytic therapy (25). In our MMD cohort, 65 patients were enrolled in the Matsushima scale VI group, which reflected a group of patients with MMD with intracranial hemorrhage or other atypical symptoms such as headache and dizziness. However, we did not observe patients with MMD in the Matsushima scale VI presented with lower Cav-1. Though the serum Cav-1 levels of patients with acute ischemic stroke were tremendously higher than the control group and the MMD group, patients with recurrent episode of ischemia or infarction (the Matsushima scale I–V) were comparable with that in patients in the Matsushima scale VI.

Histological investigations of the STA revealed apparently fibrocellular thickening intima (34–36), indicating an involvement of extracranial arteries of MMD. Moreover, the STA is relatively easy to obtain during surgery. In EDMS, the deep temporal artery (DTA) and middle meningeal artery (MMA) must be protected because of their vigorous revascularization ability after surgery. So, the tissue samples of DTA and MMA were uneasy to obtain and the STA was abundant for further immunohistochemical staining with Cav-1 antibody. We observed Cav-1 signals in the endothelial cells of intima. Except in the intima, we also detected Cav-1 expression in the smooth muscle cells of media layer, which is supposed to function in the negative remodeling of MMD arteries (8). It is widely accepted that Cav-1 was abundantly expressed in endothelial and smooth muscle cells from previous studies (37). Overexpression of Cav-1 in HMECs accelerated the capillary-like tube formation and cell migration in this study, which is consistent with previous study by Chung et al. (8). In endothelium-specific Cav-1-KO mice, angiogenesis in both the isolated aortic ring assay and hindlimb ischemia animal model was impaired (38). Similarly, in systemic Cav-1-KO mice, disrupted endothelial network formation was found in a hindlimb ischemia model (6). Mechanically, as a key structural protein of caveolae domains, Cav-1 compartmentalized endothelial nitric oxide synthase (eNOS) and exerted a tonic inhibition of eNOS activity, while excess eNOS activity secondary to Cav-1 deletion impaired endothelial angiogenesis (39). On the other hand, Cav-1 also appears to be a critical regulator of vascular endothelial growth factor (VEGF)-mediated angiogenesis by anchoring VEGF receptor (VEGFR) in the caveolae (6, 40).

There are some limitations, which should be pointed out when interpreting the results. First, in this study, we only enrolled patients with MMD who needed bypass surgery. Patients of Suzuki and Takaku's grades I and VI were excluded and only two patients of grade II were included. Patients selected under these criteria may be unable to represent the overall MMD population. Second, low mutation rate of the *RNF213* gene in Chinese Han population and the relatively small number of patients with preoperative and postoperative data weakening the strength of our conclusions. We proposed that further studies could be conducted to confirm our results, utilizing broader sample sizes, multicenter cooperation, and longer follow-up periods. Third, the number of control patients is much smaller than the MMD group, which may lead to statistical bias.

Taken together, this study demonstrated that the serum Cav-1 level of patients with MMD intermediated between the stroke group and healthy controls. The elevation of Cav-1 after bypass surgery plays an important role in the establishment of collateral circulation in patients with MMD.

## Data Availability Statement

The raw data supporting the conclusions of this article will be made available by the authors, without undue reservation.

## Ethics Statement

The studies involving human participants were reviewed and approved by Ethics Committee of Nanjing Brain Hospital. The patients/participants provided their written informed consent to participate in this study.

## Author Contributions

JZ and JM designed this study. JZ and YZ wrote this manuscript. JZ and ZY revised the manuscript. ZY and CQ performed MRI and collected data. GZ and LC performed experiments and analyzed data. SH and JM recruited the patients. All authors contributed to the article and approved the submitted version.

## Funding

This study was supported by the National Natural Science Foundation of China (Grant No. 81301049), the Natural Science Foundation of Jiangsu Province of China (Grant No. BK20130085), Jiangsu Provincial Medical Youth Talent (QNRC2016048), and the Training Project for Young Talents of Nanjing Brain Hospital.

## Conflict of Interest

The authors declare that the research was conducted in the absence of any commercial or financial relationships that could be construed as a potential conflict of interest.

## Publisher's Note

All claims expressed in this article are solely those of the authors and do not necessarily represent those of their affiliated organizations, or those of the publisher, the editors and the reviewers. Any product that may be evaluated in this article, or claim that may be made by its manufacturer, is not guaranteed or endorsed by the publisher.
